# Streptococcus pneumoniae Coinfection in COVID-19 in the Intensive Care Unit: A Series of Four Cases

**DOI:** 10.1155/2022/8144942

**Published:** 2022-08-12

**Authors:** Shrey Shah, Chaitanya Karlapalem, Pratik Patel, Nikhil Madan

**Affiliations:** ^1^Division of Pulmonary and Critical Care Medicine, Newark Beth Israel Medical Center, Newark, NJ 07112, USA; ^2^Department of Internal Medicine, Mercy Hospital, St. Louis, Missouri, USA

## Abstract

Bacterial coinfections in patients infected with SARS-CoV-2 pneumonia are uncommon, when compared to coinfections with other respiratory viruses. For example, the prevalence of bacterial coinfections in hospitalized seasonal influenza patients can exceed 30%, whereas the prevalence of bacterial coinfections in SARS-CoV-2 infection is less than 4%. Bacterial coinfections increase the severity of respiratory viral infections and have been associated with higher mortality and morbidity. Current literature shows that diagnostic testing and antibiotic therapy for bacterial infections are not necessary upon admission in majority of patients with SARS-CoV-2 patients. It is however important for the clinician to be cognizant of these coinfections since missing the diagnosis may pose a substantial risk to vulnerable COVID-19 patients. In that light, we present four cases of Streptococcus pneumoniae coinfections complicating confirmed SARS-CoV-2 infections.

## 1. Introduction

Since December 2019, the SARS-CoV-2 pandemic has spread from its epicenter in Wuhan, China, to infect over 370 million people worldwide, with over 5 million deaths associated with the disease. Bacterial coinfection is a dreaded complication of many viral respiratory tract infections with increased risk of shock and respiratory failure, prolonged ICU length of stay, and mortality [1]. In the 1918 pandemic, bacterial coinfection was a significant contributor in nearly all influenza deaths with *S. pneumoniae*, *β*-hemolytic streptococci, *S. aureus*, and *H. influenzae* as the most common pathogens [2]. Bacterial coinfections were also common in the 2009 influenza pandemic, with 55% of published autopsy series documenting a coinfection [2, 3]. With the current pandemic however, literature has shown that the prevalence of bacterial coinfections in patients infected with SARS-CoV-2 to be less than 4% [4]. The clinical significance of recognizing bacterial coinfections with SARS-CoV-2 pneumonia is established, and with the yield of diagnostic testing being low, it can be easily missed. We describe four cases of SARS-CoV-2 infection complicated by Streptococcus pneumoniae coinfection along with their presentation, radiographic changes, and their outcomes.

## 2. Case 1

**Figure 1:**
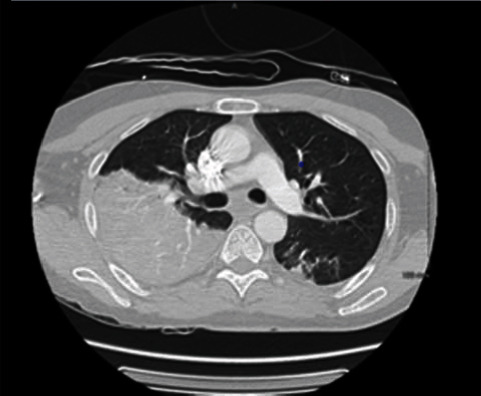
CT scan: right lower lobe consolidation.

A 51-year-old male with no past medical history came to the emergency room with complaints of shortness of breath. He was found to be hypoxemic and saturating 78% on room air and was admitted to intensive care unit for hypoxemic respiratory failure secondary to COVID-19 pneumonia. He was found to have leukopenia (2.2 × 10^3^/mcL), lymphopenia (4%), elevated ferritin of 3103 ng/mL, and elevated D-dimer of 3.42 mg/L. He was found to have *S. pneumoniae* pneumonia and bacteremia on cultures sent out on admission. Computed tomography of the chest showed multifocal pneumonia (see Figure 1). He was tachycardic and tachypneic despite support with high flow nasal cannula, and he was intubated for further support within 24 hours of admission. He was treated with remdesivir and dexamethasone for his COVID-19 infection. He received ceftriaxone for his streptococcus pneumonia. His course was complicated by ventilator-associated pneumonia from ESBL Klebsiella pneumonia and was started on ertapenem. Patient went into septic shock and died from the complications after a prolonged ICU course of 24 days.

## 3. Case 2

**Figure 2:**
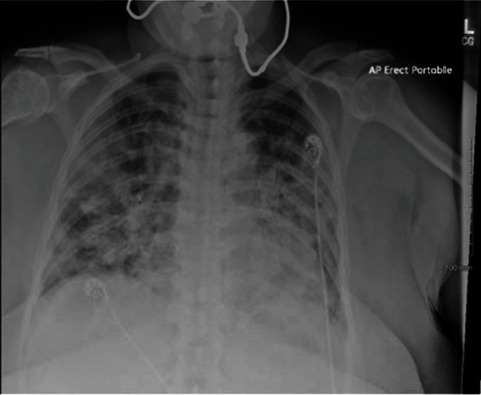
Chest X-ray: bilateral consolidations.

A 63-year-old female with no past medical history came to the emergency room with complaints of shortness of breath, cough, and sputum production. She tested positive for COVID-19 infection 7 days before presentation. Patient was found to be hypoxemic and saturating at 50% on room air and was also tachypneic. Laboratory tests revealed leukocytosis of 19.3 × 10^3^/mcL, lymphopenia (6%), ferritin of 915 ng/mL, D-dimer of 3.92 mg/L, and procalcitonin of 0.60 ng/mL. Chest X-ray showed bilateral patchy consolidations (see Figure 2). She was started on high-flow nasal cannula and admitted to the intensive care unit for management of her acute hypoxemic respiratory failure. Her Streptococcus pneumonia urine antigen came back positive. She was also found to have deep vein thrombosis on venous duplex done 2 days after admission. She was treated with remdesivir, dexamethasone, and tocilizumab for COVID-19 infection. She was treated with ceftriaxone for her Streptococcus pneumonia and Lovenox for her deep vein thrombosis. She was weaned off oxygen support over next 3 weeks, and she was discharged home on home oxygen.

## 4. Case 3

**Figure 3:**
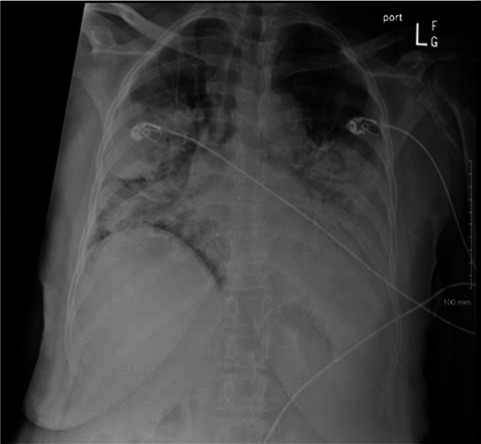
Chest X-ray: bilateral consolidations.

A 64-year-old female with a past medical history of hypertension came with 8-day history of fever, cough, and shortness of breath. Patient was found to be hypoxemic, tachypneic, and tachycardic in the emergency room. She was intubated and admitted to the hospital for management of her acute respiratory distress syndrome. Her Streptococcus pneumonia urine antigen was found to be positive. Her laboratory tests revealed normal white blood cell count, ferritin of 380 ng/mL, D-dimer of 3.494 *μ*g/mL, and procalcitonin of 8.10 ng/mL. Chest X-ray performed showed bilateral consolidations (see Figure 3). Patient was treated with remdesivir and dexamethasone for her COVID-19 infection. Patient was treated with 7-day course of cefepime for her Streptococcus pneumonia. Her ARDS was managed with lung protective ventilation, paralytics, and nitric oxide. Patient was weaned off nitric oxide, paralytics, and mechanical ventilator. Patient was extubated on the 18th day to BiPAP and slowly transitioned to nasal cannula. She was optimized over the next few days and discharged to subacute rehab.

## 5. Case 4

**Figure 4:**
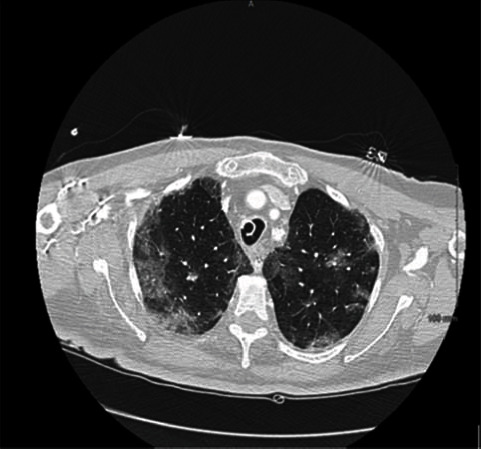
CT scan: bilateral ground glass opacities.

An 87-year-old female with a past medical history of hypertension, diabetes mellitus, hyperlipidemia, breast cancer (on letrozole), and diverticulosis was brought to the hospital with altered mental status and shortness of breath. In the emergency room, patient was hypoxic with labored breathing and was intubated. Her laboratory tests revealed white blood cell count of 9.9 × 10^3^/mcL, D-dimer of 2.23 mg/L, and procalcitonin of 6.84 ng/mL. Computed tomography of the chest showed diffuse bilateral patchy ground glass infiltrates (see Figure 4). Patient was found to be COVID-19 positive, and her sputum cultures came back positive for Streptococcus pneumoniae. Patient was treated with remdesivir and dexamethasone for her COVID-19 infection. Patient was treated with ceftriaxone for her Streptococcus pneumonia. Patient was extubated 2 weeks later but went into respiratory failure requiring reintubation. Patient got a tracheostomy and feeding tube placement. She had a prolonged stay in the hospital for a month. She was weaned to tracheostomy collar and transferred to a rehabilitation facility.

## 6. Discussion

In the series of four cases described above, the median age for cases is 63.5 with age ranges from 51 to 87 years. The median number of comorbidities is 0.5, with hypertension being the most common. All four patients had some shortness of breath on presentation. Two patients had cough on presentation out of which one presented with dry cough and other one had cough with sputum production. According to one report, 50% of patients with SARS-CoV-2 infection have productive cough, and as such, productive cough is not a unique finding for bacterial pneumonia [5]. Lab findings showed leukocytosis in one patient, normal white cell count in two patients, and leukopenia in one patient. Inflammatory markers were elevated in all patients including elevated ferritin and D-dimer in all patients and elevated procalcitonin in 3 patients. Average hospital length of stay was 26.75 days, average ICU stay was 21.75 days, and average ventilator days were 19.25 days (see Table 1).

All patients had abnormalities noted on their imaging study. One of them had multifocal consolidations noted on their CT chest. Two of them had bilateral consolidations, and the last patient had bilateral ground glass opacities. The classic radiologic feature of S. pneumoniae is a consolidative lobar pneumonia with some studies showing various radiological patterns including patchy “bronchopneumonia” pattern, interstitial pattern, and mixed patterns [6].

Streptococcal infection was detected either through urine antigen test (three patients had positive urine antigen test), or sputum cultures (one had positive sputum cultures), or blood cultures. One patient had pneumococcal bacteremia while all of them had pneumococcal pneumonia. Pneumococcal bacteremia is associated with a higher in-hospital mortality and typically have a longer length of stay in the hospital compared to those with pneumococcal pneumonia [7]. One of the four patients (25%) died in this series—the 51 yr old male with no comorbidities who died from complications from septic shock from pneumococcal bacteremia.

The burden of bacterial coinfections in patients with confirmed SARS-CoV-2 infection has not been studied extensively. A meta-analysis of studies conducted in 2020 indicated a 7% of hospitalized patients with SARS-CoV-2 patients had a bacterial coinfection, and this number increased to 14% in studies that included only ICU patients [8]. It was a different picture in the influenza epidemic of 2009. 25% of severe or fatal cases of influenza A (H1N1) pdm09 had a bacterial coinfection [9]. In the near-pandemic of severe acute respiratory syndrome coronavirus (SARS-CoV) of 2003 and the Middle East respiratory syndrome coronavirus (MERS-CoV) of 2012, the frequency of bacterial coinfections was not clearly studied. There was only one study—a multicenter study of patients admitted to ICUs in Saudi Arabia that identified 19% of patients with MERS had bacterial coinfection [10]. The temporal relationship between bacterial coinfections in influenza and other novel coronaviruses in the past raises concern that bacterial coinfection could be an often-missed complication of the current SARS-CoV-2 infection as well. There are no guidelines or consensus to diagnose bacterial coinfections in patients with SARS-CoV-2 pneumonia. This makes it furthermore challenging in already overburdened hospital systems, especially as many of the features tend to overlap.

Streptococcus pneumoniae is a gram positive, facultative anaerobic organism that is the most common cause of community acquired bacterial pneumonia. S. pneumoniae predominantly affects frail populations, with major risk factors being advanced age, smoking, alcoholism, diabetes, chronic pulmonary disease, and immunodeficiency states arising from renal disease, liver disease, or HIV [11]. A similar demographic of patients has also been studied in SARS-CoV-2 infection that is associated with less favorable outcomes and prognosis. There is also a substantial overlap in the radiological features as well. Even though the classical presentation of a pneumococcal pneumonia is thought to be unilateral and consolidative, in many cases, it appears to be bilateral with an interstitial pattern. This overlap with SARS-CoV-2 pneumonia findings on radiology makes it necessary to have a high suspicion of index to diagnose a coinfection with pneumococcal pneumonia. Furthermore, there are no specific clinical features or laboratory findings to diagnose bacterial pneumonia in patients affected with SARS-CoV-2 pneumonia. A sputum culture for confirmation of *S. pneumoniae* is limited by very low sensitivity; however, the detection of the pneumococcal C-polysaccharide antigen in urine is highly sensitive and specific [12].

## 7. Conclusions

Current literature shows that the overall proportion of COVID-19 patients who have a bacterial coinfection is lower than in previous influenza pandemics [8]. Therefore, there are no current published guidelines on the role of empiric antibiotics in patients with SARS-2-CoV pneumonia. However, with this case series, we would like to highlight the possibility of a coinfection of *S. pneumoniae* with SARS-2-CoV infection to avoid misdiagnosis and delay in antibiotic therapy.

Patients should be evaluated on a case-to-case basis, especially keeping in mind that several overlapping clinical and radiological features exist between the two. If antibiotics are initiated, it is important to reevaluate the need to adjust or discontinue them based on the individual's progress.

## Figures and Tables

**Table 1:** Patient demographics and hospital stay details.

Age	Sex	Past medical history	Symptoms on presentation	Lab findings	Radiological findings	Diagnosis of S. pneumoniae	Disposition	Treatment	Outcome	Hospital length of stay	ICU length of stay	Ventilator days
51	M	None	Shortness of breath	Hypoxemia 78% on RA, leukopenia 2200/mcl 4%lymphopenia, ferritin 3103 ng/mL, D-dimer 3.42 mg/L	CT-multifocal pneumonia	Urine antigen and blood cultures for S. pneumonia-positive	ICU complicated by VAP from ESBL and Klebsiella pneumoniae	Remdesivir, dexamethasone for COVID-19. Ceftriaxone for pneumonia	Died from septic shock complications	24	24	24
63	F	None	Shortness of breath, cough, and sputum production	Hypoxemia 50% on RA, leukocytosis 19.3 k/mcl, lymphopenia 6%, ferritin 915 ng/mL, D-dimer 3.92 mg/L, procalcitonin 0.6 ng/mL	Chest X-ray-bilateral patchy consolidations	Urine antigen for S. pneumonia-positive	ICU complicated by DVT	Remdesivir, dexamethasone, tocilizumab, ceftriaxone for pneumonia, and Lovenox for DVT	Discharged home after 3 weeks	25	9	0
64	F	HTN	Fever, cough, sob, hypoxia	Normal WBC, ferritin 380 ng/mL, D-dimer 3.494 *μ*g/mL, procalcitonin 8.10 ng/mL	Chest X-ray-bilateral consolidations	Urine antigen for S. pneumoniae-positive	ICU	Remdesivir and dexamethasone for COVID-19. 7-day course of cefepime for pneumonia. ARDS-lung protective ventilation, paralytics, and nitric oxide	Discharge to subacute rehab after extubation to nasal cannula	24	20	19
87	F	HTN, HLD, DM, breast cancer on letrozole and diverticulosis	AMS, sob, hypoxia	WBC 9.9 k/mcl, D-dimer 2.23 mg/L, procalcitonin of 6.84 ng/mL	CT-bilateral patchy GG infiltrates	Sputum cultures-S. pneumonia	ICU	Remdesivir and dexamethasone for COVID-19. Ceftriaxone for S. pneumonia	Extubated→respiratory failure→reintubated. Tracheostomy and feeding tube and transferred to rehab	34	34	34
